# Proximal Tubule Epithelial Cell Specific Ablation of the Spermidine/Spermine N^1^-Acetyltransferase Gene Reduces the Severity of Renal Ischemia/Reperfusion Injury

**DOI:** 10.1371/journal.pone.0110161

**Published:** 2014-11-12

**Authors:** Kamyar Zahedi, Sharon Barone, Yang Wang, Tracy Murray-Stewart, Prabir Roy-Chaudhury, Roger D. Smith, Robert A. Casero, Manoocher Soleimani

**Affiliations:** 1 Division of Nephrology and Hypertension, Department of Internal Medicine, University of Cincinnati College of Medicine, Cincinnati, Ohio, United States of America; 2 The Sidney Kimmel Comprehensive Cancer Center, Johns Hopkins University School of Medicine, Baltimore, Maryland, United States of America; 3 Department of Pathology and Laboratory Medicine, University of Cincinnati College of Medicine, Cincinnati, Ohio, United States of America; 4 Veterans Affair Medical Center, Cincinnati, Ohio, United States of America; Temple University, United States of America

## Abstract

**Background:**

Expression and activity of spermidine/spermine N^1^-acetyltransferase (SSAT) increases in kidneys subjected to ischemia/reperfusion (I/R) injury, while its ablation reduces the severity of such injuries. These results suggest that increased SSAT levels contribute to organ injury; however, the role of SSAT specifically expressed in proximal tubule epithelial cells, which are the primary targets of I/R injury, in the mediation of renal damage remains unresolved.

**Methods:**

Severity of I/R injury in wt and renal proximal tubule specific SSAT-ko mice (PT-SSAT-Cko) subjected to bilateral renal I/R injury was assessed using cellular and molecular biological approaches.

**Results:**

Severity of the loss of kidney function and tubular damage are reduced in PT-SSAT-Cko- compared to wt-mice after I/R injury. In addition, animals treated with MDL72527, an inhibitor of polyamine oxidases, had less severe renal damage than their vehicle treated counter-parts. The renal expression of HMGB 1 and Toll like receptors (TLR) 2 and 4 were also reduced in PT-SSAT-Cko- compared to wt mice after I/R injury. Furthermore, infiltration of neutrophils, as well as expression of tumor necrosis factor-α (TNF-α), monocyte chemoattractant protein-1 (MCP-1) and interleukin-6 (IL-6) transcripts were lower in the kidneys of PT-SSAT-Cko compared to wt mice after I/R injury. Finally, the activation of caspase3 was more pronounced in the wt compared to PT-SSAT-Cko animals.

**Conclusions:**

Enhanced SSAT expression by proximal tubule epithelial cells leads to tubular damage, and its deficiency reduces the severity of renal I/R injury through reduction of cellular damage and modulation of the innate immune response.

## Introduction

Polyamines (spermidine, Spd and spermine, Spm) are aliphatic cations that interact with nucleic acids and proteins. Through their interactions, polyamines play important roles in maintenance of nucleic acid structure, gene transcription, signal transduction and cell proliferation [1]–[5]. The cellular levels of polyamines are tightly regulated through their import, export, synthesis and catabolism. Polyamines are catabolized by back-conversion through their stepwise acetylation and oxidation by spermidine/spermine N^1^-acetyltransferase (SSAT) and N^1^-acetylpolyamine oxidase (APAO), respectively, or oxidation of Spm by spermine oxidase (SMO). Oxidation of acetylated Spm and Spd by APAO generates H_2_O_2_ and 3-acetoaminopropanal, whereas oxidation of Spm by SMO generates H_2_O_2_, 3-aminopropanal. Both H_2_O_2_ and the respective aldehydes can lead to cell injury.

The expression and activity of SSAT increases in organs (e.g. liver, kidney and brain) subjected to ischemia/reperfusion (I/R), sepsis, toxic and traumatic injuries [6]–[10]. Transgenic animals that express high levels of SSAT develop skin lesions and pancreatitis [11]–[13]. *In vitro*, expression of SSAT causes oxidative stress, DNA damage, cell cycle arrest, and cell death [14]–[16]. These results suggest that elevated SSAT levels contribute to the onset of cell damage and tissue injury. Using SSAT-ko mice, we have shown that SSAT deficiency reduces the severity of sepsis and I/R induced injuries [6], [17]. Although the aforementioned studies indicate that SSAT plays a maladaptive role in renal injuries, they do not identify the specific cells that are the source of this enzyme. We hypothesize that increased SSAT expression and activity in renal proximal tubule cells in response to renal I/R injury contributes to tubular damage and kidney dysfunction. In order to test this hypothesis, we generated proximal tubule cell-specific SSAT knockout (PT-SSAT-Cko) mice and determined the impact of cell specific ablation of the SSAT gene on the severity of kidney I/R injury.

## Materials and Methods

### Reagents

All chemicals were purchased from Sigma-Aldrich (St. Louis, MO) unless otherwise indicated. Oligonucleotides were purchased from Invitrogen (Carlsbad, CA). The following antibodies were used in this study: Rabbit anti-actin (Santa Cruz Biotech, Santa Cruz, CA), Rabbit anti-pro and cleaved Caspase 3 (H-277, Santa Cruz Biotech, Santa Cruz, CA), Rabbit anti-cleaved caspase 3 (Sigma-Aldrich, St Louis, MO), Rabbit anti-Poly (ADP-ribose) polymerase 1/2 (Santa Cruz Biotech, Santa Cruz, CA), Rabbit anti toll-like receprtor 2 (TLR2; Santa Cruz Biotech, Santa Cruz, CA) and 4 (TLR4; Santa Cruz Biotech, Santa Cruz, CA) and rabbit anti-HMGB1 (Novus Biologicals, Littelton, CO). All secondary antibodies were purchased from Invitrogen.

### Generation and genotyping of PT-SSAT-Cko mice

**Figure 1:**
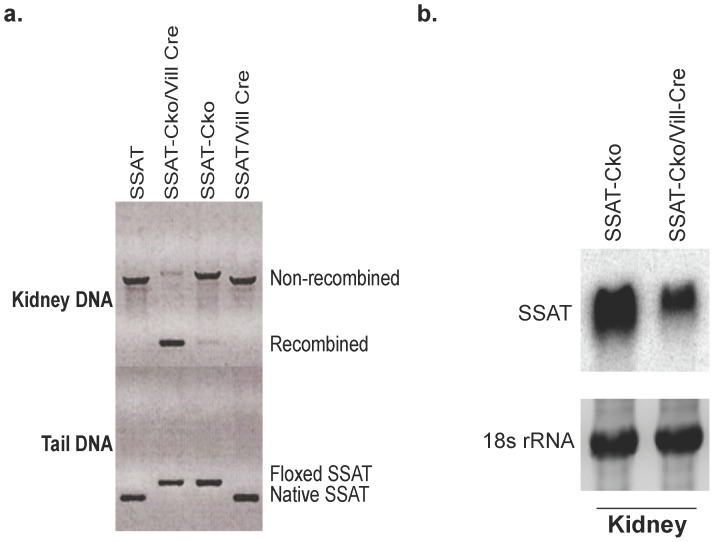
Characterization of proximal tubule cell specific SSAT knockout mice. a) Disruption of the SSAT gene was confirmed by examining the PCR amplification products of genomic DNA from the tail (bottom panel) and kidney (top panel) of SSAT-Cko/Vill-Cre (PT-SSAT-Cko) and their Cre-deficient (SSAT-Cko) littermates. b) Kidney RNA (30 µg/well) from PT-SSAT-Cko and wt mice was size fractionated and subjected to northern blot analysis in order to assess the effect of cre mediated ablation of the SSAT gene on the expression of its mRNA.

LoxP-SSAT (C57BL6-SSAT-Cko^Neo-/Flp-^) mice were generated in our laboratory [8]. Proximal tubule specific SSAT deficient (PT-SSAT-Cko) mice were generated by cross breeding of the C57BL6-SSAT-Cko^Neo-/Flp-^ with C57BL6-Villin-promoter driven Cre-recombinase transgenic mice (Jackson Laboratories, Bar Harbor, ME). The Cre-recombinase mediated disruption of the SSAT gene was confirmed by comparing the genomic DNA from the tail and kidney as previously described [8]. Amplified DNA was examined for the presence of ∼1450bp (wt) and ∼240 bp (SSAT-Cko) products (**Fig. 1a**). The effect of SSAT gene ablation on the expression of its mRNA in the kidney was examined by northern-blot analysis (**Figs. 1b**).

### Induction of renal I/R injury

Male PT-SSAT-Cko and wt mice (28–32 g) were used in these studies. Animals were provided with ample food and water and kept on a 12hr–12hr light–dark cycle at all times. In these studies, animals (n = 8/experimental group) were subjected to renal I/R injury through bilateral clamping of renal arteries for 30 minutes or sham operation as previously described [17]. Briefly, animals were anesthetized by intraperitoneal (i.p.) injection of Ketamine: Xylazine solution (100∶10 µg/g body weight). This anesthetic regiment was used instead of inhaled gas since it is not associated with the induction of hypoxia. Once animals were unconscious the site of surgery (abdomen) was shaved and swabbed with betadine solution and ethanol. A medial incision was made, kidneys were exposed and animals were subjected to renal I/R injury through bilateral clamping of renal arteries for 30 minutes. As uninjured controls animals were subjected to sham operation (kidneys were not clamped). At timed intervals (12, 24, 48 and 72 hours), animals were sacrificed through administration of pentobarbital (100 mg/g i.p.). Blood was collected by direct cardiac puncture and processed to obtain serum. Kidneys were harvested and either fixed in 4% paraformaldehyde, preserved in 70% ethanol and processed for histology and immunofluorescent studies, or snap-frozen in liquid nitrogen and used for RNA and protein extraction. Experiments assessing the effect of the inhibition of polyamine oxidases on the severity of renal I/R injury were performed as outlined above except wt animals (n = 5/group) were treated with vehicle (saline) or polyamine oxidase inhibitor, MDL72527 (100 mg/kg i.p.), 30 minutes after surgery. All studies involving animal were performed according to the standards in the “Guide for the Care and Use of Laboratory Animals” following a protocol approved by the University of Cincinnati Animal Care and Use Committee. All animal work complied with the National Institutes of Health guidelines.

### Assessment of kidney histopathology

The histology of kidneys from injured and sham operated wt and PT-SSAT-Cko mice (minimum of 3 animals per group) was compared by light microscopic examination of H&E-stained sections in a blinded manner. Briefly, paraformaldehyde fixed/ethanol preserved tissue samples in were paraffin embedded. Tissue sections (5 µm) were cut, stained with H&E, slides were assigned random numerical codes for blinded evaluation and examined at 100 and 200X magnification. The extent of renal injury was assessed by examining the cortical and corticomedullary regions of the kidney for tubular dilatation, interstitial edema, cast formation and leukocyte infiltration. Kidneys were assigned an injury score of 0 to 3 (0, no injury; 1, mild injury; 2, moderate injury; 3, severe injury) to compare the severity of tissue damage.

### Assessment of renal function

Serum creatinine and blood urea nitrogen (BUN) levels were measured using commercially available kits following the manufacturers (Bioassay Systems, Hayward, CA) instructions.

### Measurement of tissue polyamine levels

Polyamine pools were analyzed chromatographically as described previously [18], [19].

### Cell culture studies

Human embryonic kidney cell line, HEK-293, capable of tetracycline inducible expression of SSAT was generated in our laboratory and has been described elsewhere [20]. In these studies cells treated with vehicle or tetracycline were examined for the integrity of their mitochondria using the MitoPT-JC1 mitochondrial staining kit (Immunochemistry Technologies, Bloomington, MN) or processed for protein extraction as described previously [16].

### Northern blot analysis

RNA was extracted using the Tri-Reagent protocol (Molecular Research Center, Inc. Cincinnati, OH) and subjected to northern blot analysis as previously described [10]. Equal loading of RNA samples was confirmed by assessment of the ribosomal RNA (rRNA) band intensities.

### Preparation of kidney and cell extracts

Flash frozen kidneys were pulverized, washed with ice-cold PBS and subjected to centrigugation at 7,000 g for 5 min. Supernatants were discarded and 200 µl of extraction buffer (45 mM HEPES, 0.4 M KCl, 1 mM EDTA, 0.1 mM dithiothreitol, 10% glycerol, pH 7.8) was added to each pellet. Resulting suspensions were mixed vigorously, snap frozen in liquid nitrogen and immediately thawed. Next, 30 µl of 1% Triton X-100 in extraction buffer was added to a 100 µl aliquot of each sample. Samples were mixed vigorously and incubated for 5 min on ice. After centrifugation at 14,000 g for 5 min at 4°C to remove cellular debris, the supernatants were collected. Cell extracts were prepared by lysing the harvested cells in 1X RIPA buffer. All extraction buffers were supplemented with protease and phosphatase inhibitor cocktail (Thermo Scientific, Rockford, IL). The protein contents of kidney and cell extracts were determined by BCA assay (Thermo Scientific, Rockford, IL). For analysis of protein expression levels, 30 µg of each extract was size fractionated by polyacrylamide gel electrophoresis, transferred to nitrocellulose membrane and subjected to western blot analysis as previously described [16].

### Measurement of renal cytokine levels

The expression levels of tumor necrosis-α (TNF-α), monocyte chemoattractant protein-1 (MCP-1) and interleukin-6 (IL-6) were assessed by northern blot analysis. The levels of these cytokines in kidney extracts of control and injured mice of both genotypes were measured using the appropriate ELISA kits following the manufacturers (eBioscience, San Diego, CA) instructions.

### Immunofluorescent microscopic analysis of kidney sections

Kidney sections (5 µm) were processed for immunofluorescent detection of cleaved caspase 3, TLR2 and TLR4 as described previously [21].

### Data analysis

Values are expressed as mean+/-SEM. The significance of differences between mean values of multiple samples was examined using ANOVA. A *“P”* value of less than 0.05 was considered statistically significant.

## Results

### Proximal tubule epithelium-specific ablation of the SSAT gene reduces the severity of renal I/R injury

**Figure 2:**
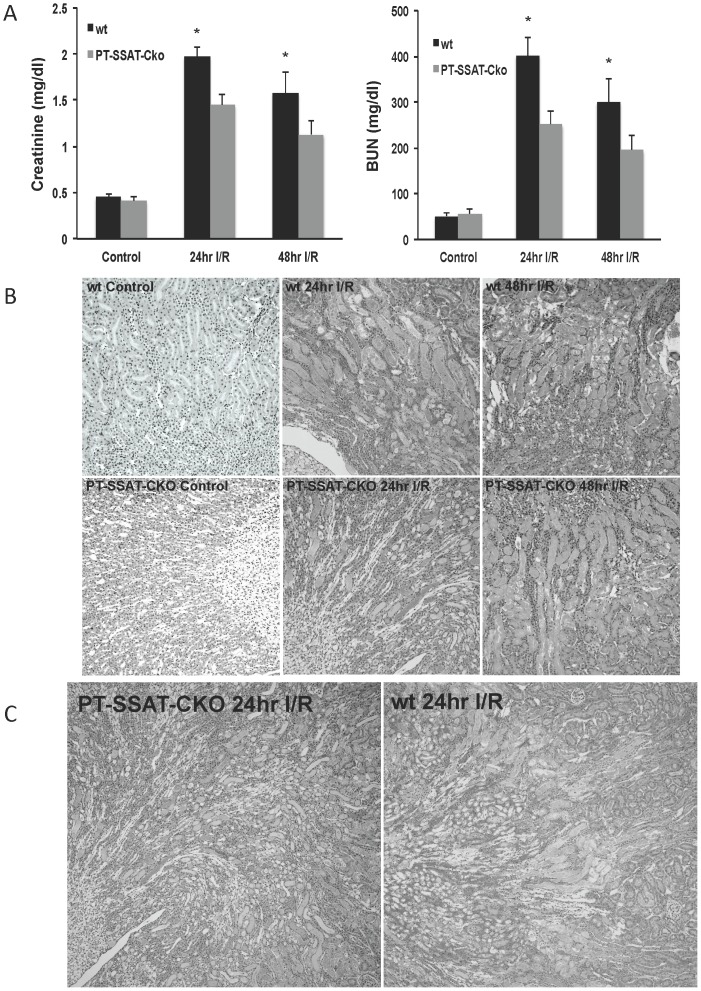
Assessing the effect of proximal tubule cell specific SSAT deficiency on the severity of renal I/R injury. Wt and PT-SSAT-Cko were subjected to Sham or I/R surgery. Animals (n = 8/experimental group) were sacrificed at timed intervals after treatment. a) Serum creatinine and BUN levels of sham operated and injured wt and PT-SSAT-Cko mice were compared following the protocol out lined in the Methods Section. Results are expressed as mean+/-SEM. A p<0.05 is considered significant. b) Kidney histology (Mag 200x) of sham operated and injured wt- and PT-SSAT-Cko- animals were compared. c) Kidney histology (Mag 100x) of injured wt- and PT-SSAT-Cko- animals was compared.

The role of proximal tubule cell specific synthesis of SSAT in I/R injury has not been previously addressed. The severity of I/R injury was compared in wt and PT-SSAT-Cko mice in order to specifically determine the role of increased expression of SSAT in proximal tubule epithelial cells in the mediation of tubular damage and kidney dysfunction. Serum creatinine and BUN levels in all injured animals were significantly elevated compared to sham-operated animals (**Fig. 2a**). However, at 24 and 48 hours post I/R injury the serum creatinine and BUN levels were significantly (p<0.05) lower in PT-SSAT-Cko compared to wt mice (**Fig. 2a**). Next, in order to confirm the results of the aforementioned functional studies, we examined the histology of kidneys in wt and PT-SSAT-Cko mice after sham operation or renal I/R injury. Examination of the superficial cortical (outer-cortex) and cortico-medullary (inner-cortex/outer-medulla) regions of the kidneys of sham-operated (non-ishemic) wt- and PT-SSAT-Cko mice did not reveal any histological abnormalities (**Table 1**; **Fig. 2b**). Comparison of the cortical histopathology of ischemic kidneys revealed that wt mice showed moderate tubular dilatation, interstitial edema, and cast formation, the PT-SSAT-Cko mice were protected against these changes and had reduced injury levels compared to their wt counterparts (**Fig. 2b**). Examination of the histology of corticomedullary region of animals subjected to I/R injury revealed that whereas wt animals have extensive tubular damage (e.g. severe tubular dilation and cast formation) the PT-SSAT-Cko mice display significant protection against structural damage (e.g. moderate levels of tubular dilatation, cast formation and inflammatory cell infiltration) relative to their wt littermates (**Fig. 2b**). In general the SSAT-deficient animals exhibited less extensive and less severe ischemic injury compared to wt animals (**Table 1** **,** **Fig. 2c**). Our results suggest that the severity of parenchymal damage is reduced in PT-SSAT-Cko animals. The reduction of the severity of kidney damage (i.e. renal histopathology) in PT-SSAT-Cko animals in addition to the data indicating that these animals have better preserved renal function (i.e. serum creatinine and BUN levels) indicates that proximal tubule specific ablation of SSAT gene protects the kidneys against I/R injury. By extension, these studies suggest that enhanced SSAT expression and the attendant increase in polyamine back-conversion in proximal tubular epithelial cells plays a maladaptive role in the injury process in the kidneys subjected to I/R.

**Table 1:** Renal histopathology scores for wt and PT-SSAT-Cko mice subjected to sham surgery or renal I/R injury.

			Cortex					Inner cortex/ Outer medulla		
	Tubular Dilitation	Edema	Cast	Leukocyte Infiltration	Cumulative Score	Tubular Dilitation	Edema	Cast	Leukocyte Infiltration	Cumulative Score
**Wild Type Sham (n = 2)**	0.0+/−0.0	0.0+/−0.0	0.0+/−0.0	0.0+/−0.0	0.0+/−0.0	0.0+/−0.0	0.0+/−0.0	0.0+/−0.0	0.0+/−0.0	0.0+/−0.0
**PT-SSAT-Cko Sham (n = 2)**	0.0+/−0.0	0.0+/−0.0	0.0+/−0.0	0.0+/−0.0	0.0+/−0.0	0.0+/−0.0	0.0+/−0.0	0.0+/−0.0	0.0+/−0.0	0.0+/−0.0
**Wild Type 24hr I/R (n = 3)**	1.3+/−0.5	0.3+/−0.6	1.0+/−0.0	0.0+/−0.0	2.7+/−0.5*	3.0+/−0.0*	0.7+/−0.5	3.0+/−0.0*	1.3+/−0.5	8.0+/−0.8
**PT-SSAT-Cko 24hr I/R (n = 3)**	1.0+/−0.0	0.0+/−0.0	1.0+/−0.0	0.0+/−0.0	2.0+/−0.0	2.3+/−0.5	1.0+/−0.0	2.3+/−0.5	0.7+/−0.9	6.3+/−1.9
**Wild Type 48hr I/R (n = 3)**	1.0+/−0.0	0.0+/−0.0	0.7+/−0.5	0.0+/−0.0	1.7+/−0.5	2.3+/−0.5	0.7+/−0.5	3.0+/−0.0	1.0+/−0.0	7.0+/−0.8
**PT-SSAT-Cko 48hr I/R (n = 3)**	0.7+/−0.9	0.3+/−0.5	0.8+/−1.2	0.7+/−0.9	2.5+/−3.3	1.7+/−1.2	0.5+/−0.4	2.0+/−1.4	1.3+/−1.2	5.5+/−3.9

*p<0.05.

**Figure 3:**
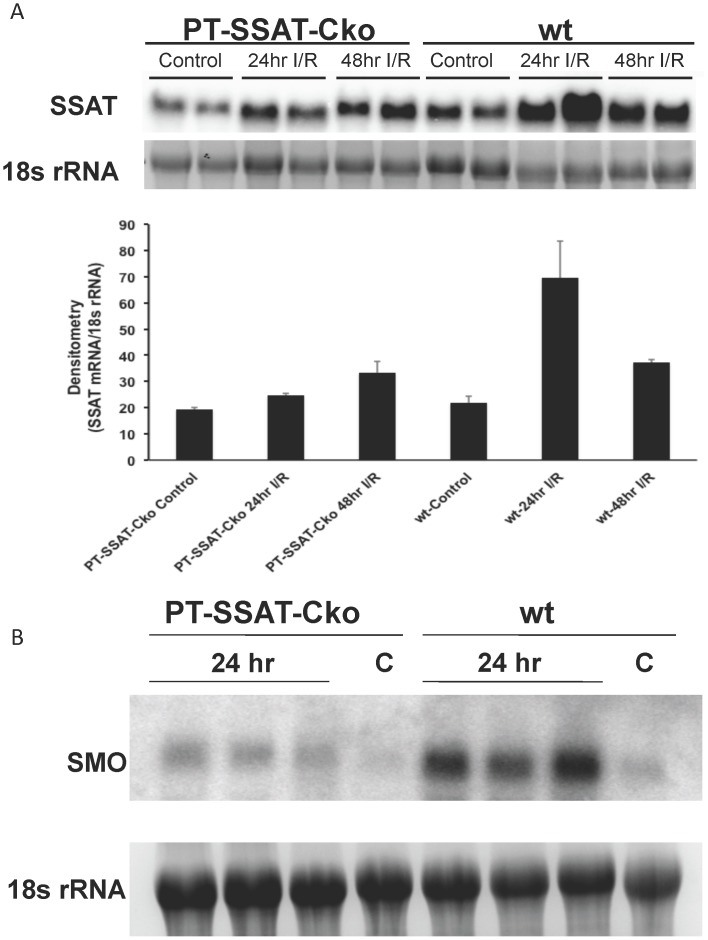
Comparison of the time course of expression of SSAT and SMO in the kidneys of sham operated and injured wt- and PT-SSAT-Cko-mice. a) Kidney RNA (30 µg/well) from sham operated and injured wt- and PT-SSAT-Cko-mice (n = 2) was size fractionated and subjected to northern blot analysis in order to compare the expression levels of SSAT transcripts. The intensity of SSAT mRNA and 18s rRNA bands were determined by densitometry. The intensity of SSAT bands were normalized against that of the corresponding 18s rRNA bands. The graph depicts the average normalized SSATmRNA/18s rRNA values. b) Kidney RNA (30 µg/well) from sham operated (n = 1) and injured wt and PT-SSAT-Cko mice (n = 3) was size fractionated and subjected to northern blot analysis in order to compare the SMO mRNA levels.

**Figure 4:**
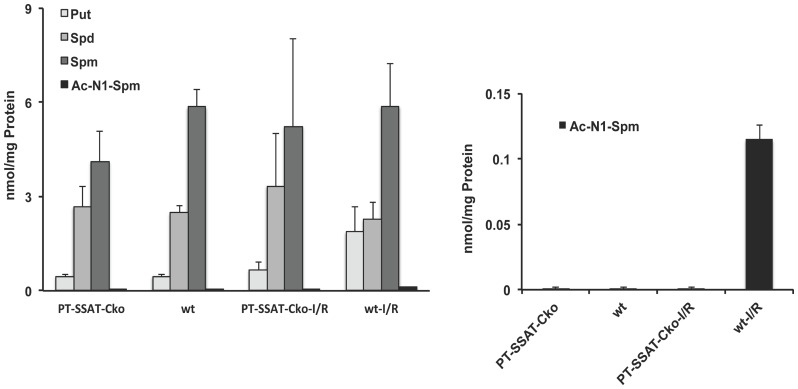
Measurement of polyamine levels in the kidneys of wt and PT-SSAT-Cko mice subjected to sham surgery or renal I/R injury. Kidney polyamine contents of wt and PT-SSAT-Cko mice were measured by HPLC. a) Kidney Put, Spd and Spm levels were determined at 24 hours post-sham or I/R surgery in wt and PT-SSAT-Cko animals. b) Acetyl-N^1^-spermine levels in the kidneys of sham operated and injured wt and PT-SSAT-Cko mice were compared.

Next, we compared the expression of SSAT and SMO in sham-operated and injured wt and PT-SSAT-Cko mice. Our results indicate that SSAT mRNA expression increases in the kidneys of wt animals after I/R injury (24 hour post injury, **Fig. 3a**). In contrast, the renal expression of SSAT mRNA in PT-SSAT-Cko animals subjected to I/R injury did not significantly differ from that of their sham operated counterparts (**Fig. 3a**). While the expression of the SMO transcript was elevated in both genotypes after I/R injury, its levels were higher in the wt compared to PT-SSAT-Cko animals (**Fig. 3b**). Comparison of kidney polyamine levels at 24 hours post I/R or sham operation **(** **Fig. 4a** **)** indicate that Spd and Spm levels were similar in sham operated wt and PT-SSAT-Cko mice (2.48+/−0.23 and 5.86+/−0.56 and 2.68+/−0.63 and 4.12+/−0.95 nmol/mg protein, respectively) and did not change after I/R injury in either genotype (2.27+/−0.56 and 5.88+/−1.35 and 3.34+/−1.68 and 5.24+/−3.53 nmol/mg protein, respectively). The Put levels were similar in the kidneys of sham-operated wt and PT-SSAT-Cko mice (0.46+/−0.07 and 0.46+/−0.05 mol/mg protein). The kidney Put levels in injured PT-SSAT-Cko mice (0.67+/−0.26 nmol/mg protein) was not different from those of the sham-operated animals, while the renal Put content of wt mice after I/R injury (1.83+/−0.80) was higher than sham operated-mice of either genotype and injured PT-SSAT-Cko mice. As shown in **Fig. 4b**, acetylated polyamine levels were below detection limits in sham operated and injured PT-SSAT-Cko mice while acetylated-Spd and Spm levels were elevated in the kidneys of wt animals after I/R injury.

**Figure 5:**
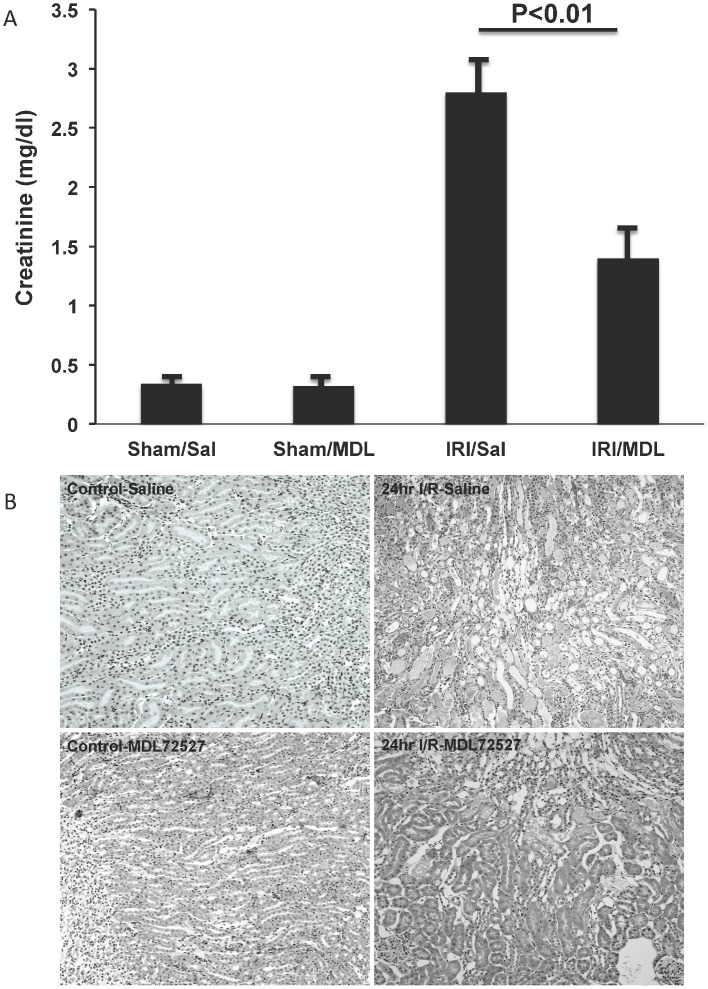
Assessing the effect of inhibition of polyamine oxidases on the severity of renal I/R injury. a) Serum creatinine levels of sham operated and injured mice injected with MDL72527 or vehicle (n = 5/experimental group) were compared following the protocol out lined in the Methods Section. Results are mean+/-SEM of three independent samples. A p<0.05 is considered significant. b) Kidney histology (Mag 200x) of control and injured animals from vehicle and MDL72527 treated groups were compared.

Reduced induction of SMO mRNA in the kidneys of PT-SSAT-Cko mice after I/R injury (**Fig. 3**) as well as the absence of acetylated polyamines that are generated via SSAT activity and are degraded by APAO to generate H_2_O_2_ and aminoaldehydes suggest that oxidation of polyamines, through the activity of both SMO and APAO, plays a role in the reduction of severity of renal I/R injury in PT-SSAT-Cko animals. In order to address the role of polyamine oxidation in renal I/R injury, we examined the effect of inhibition of polyamine oxidases with MDL72527 on the severity of tissue damage in animals subjected to renal I/R injury. Our results indicate that serum creatinine levels (1.45+/−0.2 vs. 2.8+/−0.3 mg/dl) and the severity of tubular injury (i.e. tubular dilatation, cast formation and leukocyte infiltration) were significantly reduced in the MDL72527- compared to vehicle-treated animals at 24 hours post I/R injury (**Table 2** and **Fig. 5a and b**). These results indicate that polyamine oxidation contributes to the induction of tubular damage and the loss of renal function in I/R injury.

**Table 2:** Renal histopathology scores for saline or MDL72527 treated mice subjected to Sham operation or renal I/R injury.

			Cortex					Inner cortex/ Outer medulla		
	Tubular Dilitation	Edema	Cast	Leukocyte Infiltration	Cumulative Score	Tubular Dilitation	Edema	Cast	Leukocyte Infiltration	Cumulative Score
**Saline Sham (n = 2)**	0.0+/−0.0	0.0+/−0.0	0.0+/−0.0	0.0+/−0.0	0.0+/−0.0	0.0+/−0.0	0.0+/−0.0	0.0+/−0.0	0.0+/−0.0	0.0+/−0.0
**MDL72527 Sham (n = 2)**	0.0+/−0.0	0.0+/−0.0	0.0+/−0.0	0.0+/−0.0	0.0+/−0.0	0.0+/−0.0	0.0+/−0.0	0.0+/−0.0	0.0+/−0.0	0.0+/−0.0
**Saline 24hr I/R (n = 3)**	1.4+/−0.5	0.6+/−0.8	1.3+/−0.4	0.0+/−0.0	3.2+/−1.5	2.8+/−0.4	0.6+/−0.5	3.0+/−0.0*	1.0+/−0.6*	7.2+/−1.2
**MDL72527 24hr I/R (n = 3)**	1.3+/−0.4	0.2+/−0.4	0.8+/−0.4	0.0+/−0.0	2.3+/−0.9	1.8+/−0.6	0.8+/−0.4	2.1+/−0.8	0.0+/−0.0	4.8+/−1.9

*p<0.05.

### Increased expression of SSAT in cultured cells leads to mitochondrial damage and apoptosis

**Figure 6:**
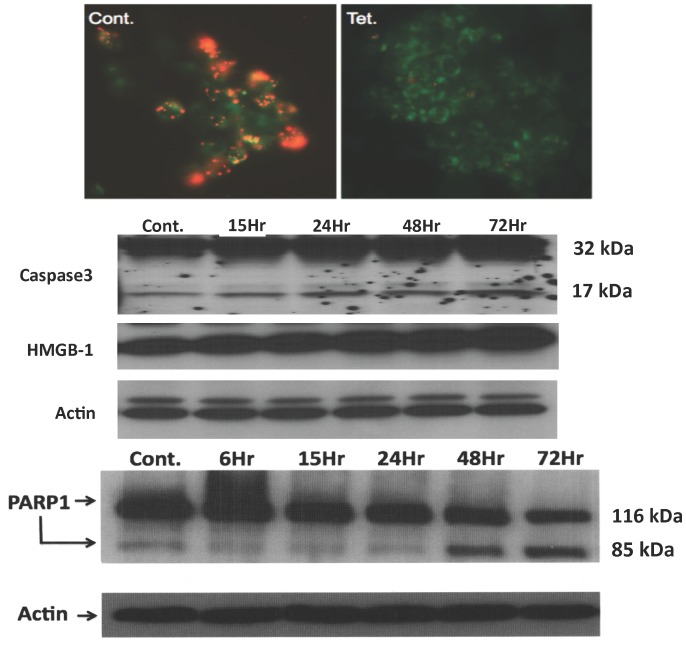
Determining the effect of SSAT expression in cultured cells. The effect of increased SSAT levels in HEK293 cells capable of tetracycline inducible expression of SSAT was examined. a) Effect of increased SSAT expression on mitochondrial integrity was compared in control (Cont.) and tetracycline-treated (Tet.), SSAT expressing, HEK293 cells by assessing the ability of these organelles to retain JC1 dye. JC1 dye is retained by non-depolarized-mitochondria (orange fluorescence) but not by depolarized-mitochondria. b) Cleavage of caspase 3 and PARP-1 (activation of apoptotic pathway), and expression of HMGB1 were examined in control (Cont.) and tetracyclin-treated, SSAT expressing, cells at timed intervals.

We have shown that increased expression of SSAT in cultured cells leads to aberrant cytoskeletal changes, DNA damage and growth arrest [16], [20]. In order to elucidate the mechanistic basis of SSAT mediated cell injury, we examined the effect of its expression on the onset of apoptosis in HEK cells capable of inducible expression of SSAT. Our results indicate that induction of SSAT leads to the loss of mitochondrial membrane potential, activation of caspase 3, and cleavage of Poly (ADP-ribose) polymerase 1 (PARP1) (**Fig. 6a and b**). In addition, induction of SSAT expression led to increased production of high mobility group B1 (HMGB1) protein (**Fig. 6b**). These results suggest that enhanced expression of SSAT in cultured cells leads to the onset of apoptosis. Furthermore, enhanced levels of HMGB1 in cells that express high levels of SSAT can lead to the activation of the innate immune response, a known contributing factor to renal I/R injury [22].

### Proximal tubule epithelial cell specific ablation of the SSAT gene modulates the onset of the innate immune response and reduces the extent of apoptotic cell death

**Figure 7:**
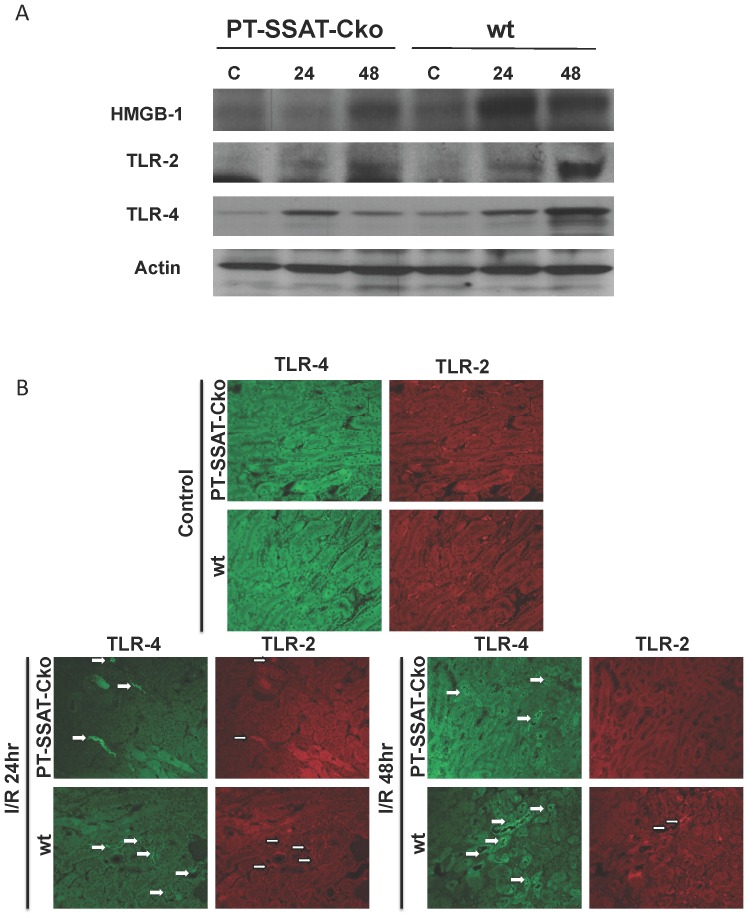
Proximal Tubule Epithelial Cell (PTEC) specific ablation of the SSAT gene dampens the onset of innate immune response and reduces the extent of I/R induced apoptotic cell death. The onset of innate immune response after I/R injury was compared in wt and PT-SSAT-Cko animals. a) Time course of the expression of HMGB1, TLR2 and 4 were compared in the kidneys of sham-operated and injured wt and PT-SSAT-Cko mice. The data are representative of three independent experiments. b) Expression of TLR2 and 4 were assessed by immunofluorescent microscopic examination of kidneys of wt and PT-SSAT-Cko mice after sham- or I/R surgery.

**Figure 8:**
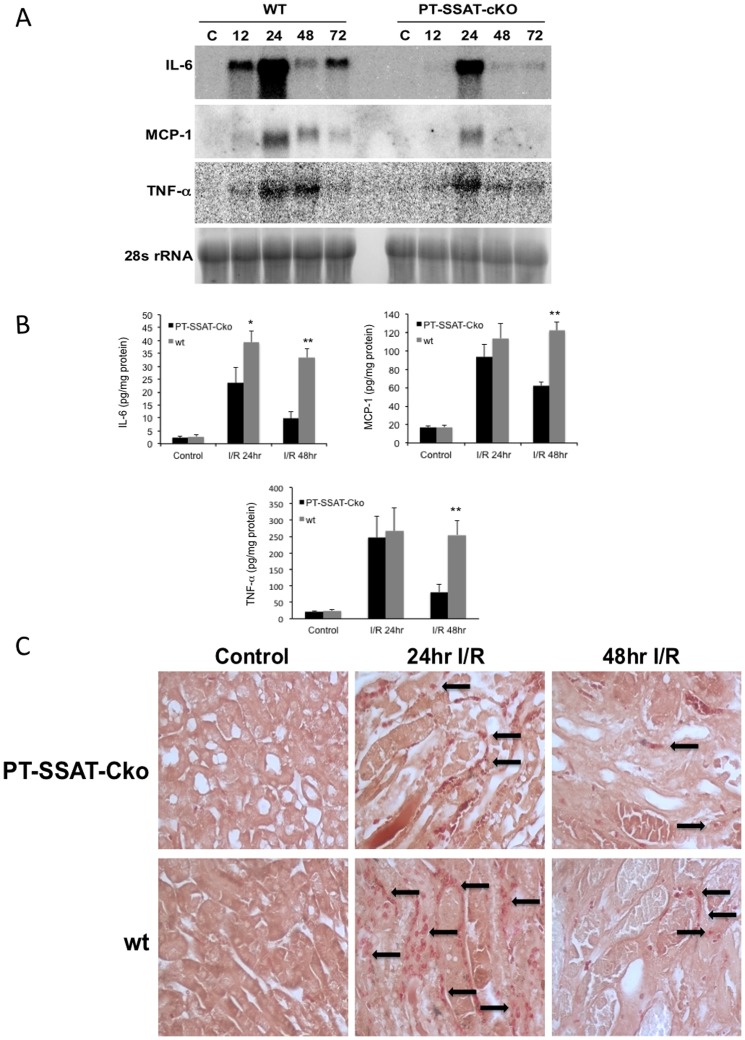
Expression of pro-inflammatory cytokines and neutrophil infiltration after I/R injury is reduced in the kidneys of PT-SSAT-Cko- compared to wt-mice. a) Kidney RNA (30 µg/well) from sham operated and injured wt and PT-SSAT-Cko mice was size fractionated and subjected to northern blot analysis in order to compare the expression levels of IL-6, MCP-1 and TNF-α transcripts. b) IL-6, MCP-1 and TNF-α levels in kidney extracts wt and PT-SSAT-Cko mice subjected to sham operation or renal I/R injury (n = 4 animals/genotype/treatment) were determined using ELISA. c) Kidney sections from sham operated and injured wt and PT-SSAT-Cko mice were subjected to chloroacetate esterase staining to examine the effect of SSAT ablation on the extent of neutrophil infiltration after renal I/R injury.

HMGB1 binding to TLR2 and 4 and activation of the innate immune response can lead to inflammation and apoptosis and play an important role in the mediation of renal I/R injury [22]–[26]. Based on increased expression of HMGB1 in SSAT expressing cells (**Fig. 6b**), and our *in vivo* results showing a reduction in leukocyte infiltration in PT-SSAT-Cko compared to wt animals (**Table 1**; **Fig. 2b and c**) we postulated that the lack of SSAT and reduced cell injury can modulate the onset of innate immunity and further reduce the severity of renal injury in PT-SSAT-Cko animals. In order to test this, we compared the onset of innate immune response (e.g. expression levels of HMGB1, TLR2 and TLR4) and apoptosis (cleaved caspase 3 levels) after I/R injury in wt and PT-SSAT-Cko mice. Although the renal expression of HMGB1, TLR2 and TLR4 increase in response to I/R injury in both groups, the expression of these molecules are reduced in PT-SSAT-Cko- compared to wt-animals (**Fig. 7 a and b**). The differences in the activation of innate immune response were further examined by comparing the extent of neutrophil infiltration and pro-inflammatory cytokine expression in the kidneys of wt and PT-SSAT-Cko animals after I/R injury. Examination of TNF-α, MCP-1 and IL-6 transcripts in the kidneys of injured animals revealed that the expression levels of these cytokines are reduced in PT-SSAT-Cko- compared to wt-mice after I/R injury (**Fig. 8a**). Assessment of the protein levels of the aforementioned cytokines also revealed that although the renal content of these cytokines is elevated in both wt- and PT-SSAT-Cko-mice, the former have a more robust response (**Fig. 8b**). Specifically, while the levels of all cytokines increased significantly in injured compared to control kidneys (sham operated animals) at 24 and 48 hours, the cytokine levels in the kidneys of wt animals compared to their PT-SSAT-Cko animals were significantly higher at 48 hrs post I/R injury (**Fig. 8b**). Additionally, chloroacetate esterase staining of the kidney sections revealed that compared to wt-mice the PT-SSAT-Cko animals have reduced levels of neutrophil infiltration (**Fig. 8c**).

**Figure 9:**
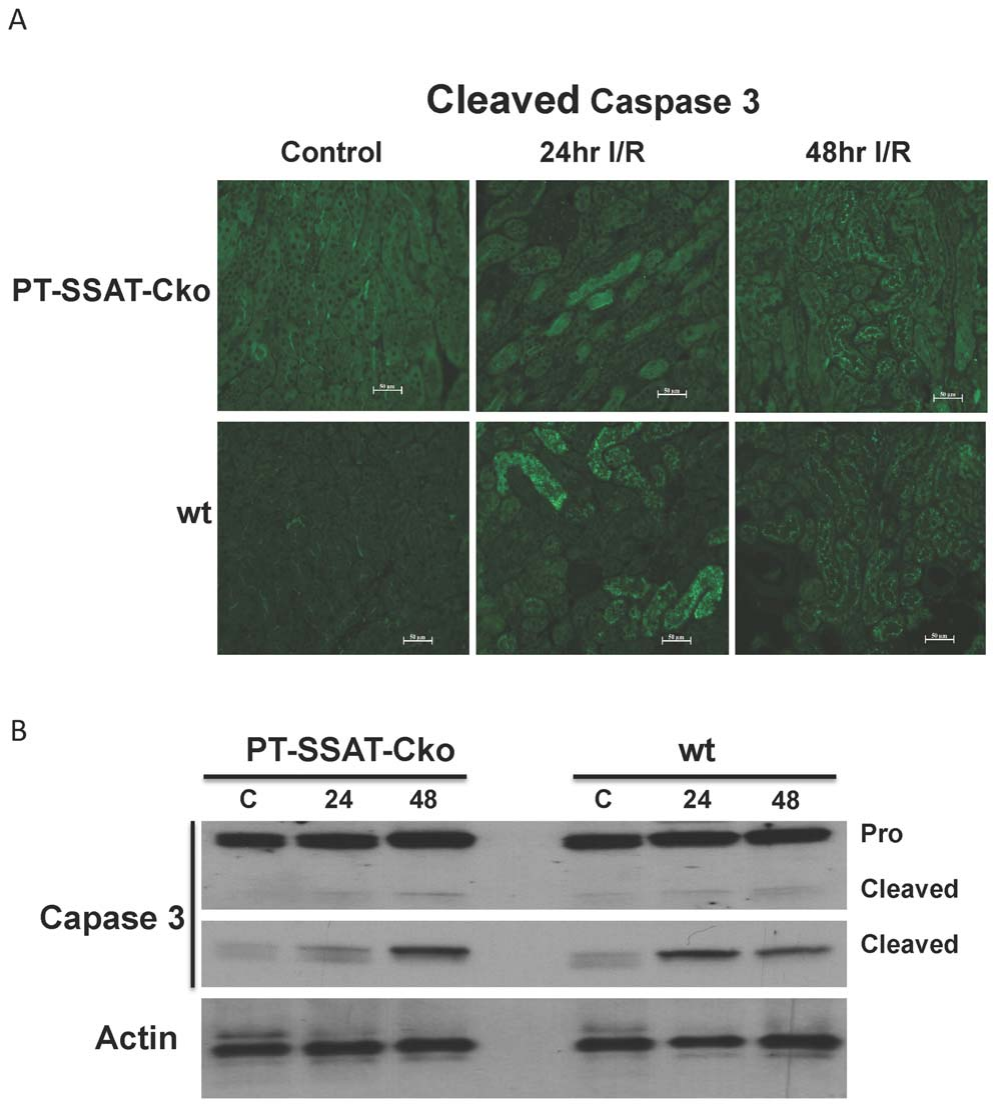
Proximal Tubule Epithelial Cell (PTEC) specific ablation of the SSAT gene reduces the extent of I/R induced apoptotic cell death. a) Activated/cleaved caspase 3 levels in the kidneys of sham operated and injured wt and PT-SSAT-Cko mice were examined by immunofluorescent microscopy (Mag 200x). b) Pro- and cleaved-caspase 3 levels in the kidneys of sham operated and injured wt and PT-SSAT-Cko mice were examined by western blot analysis (top panel: anti-pro and cleaved caspase 3; middle panel: anti cleaved-caspase 3; bottom panel: anti-actin).

Next, we examined the effect of SSAT deficiency on the onset of apoptosis, by comparing the levels of activated caspase 3 in wt and PT-SSAT-Cko animals. Activation of caspase 3 was evident in the proximal tubules of both injured wt and PT-SSAT-Cko animals. Comparison of the injured kidneys revealed that wt animals have increased levels of cleaved caspase 3 (**Fig. 9a**). Western blot analysis of kidney extracts from control and injured animals of both genotypes also confirmed the aforementioned results (**Fig. 9b** **Top panel, anti-pro and cleaved/activated caspase 3; middle panel, anti-cleaved/activated caspase 3**). These results indicate that the onset of innate immunity is modulated and the extent of renal tubule epithelial cell apoptosis is reduced in PT-SSAT-Cko animals compared to their wt littermates.

## Discussion

The expression of SSAT increases dramatically in kidneys subjected to I/R injury [10]. Enhanced expression of SSAT in cultured cells leads to the depletion of polyamines, DNA damage and growth arrest [16], [20]. Also, the extent of renal damage after I/R and bacterial lipopolysaccharide-induced renal injury is significantly reduced in SSAT-ko mice [6], [17]. Although, these results suggest that increased SSAT expression and elevated polyamine back-conversion are critical in the mediation of I/R- and LPS-induced renal injuries, the role of increased expression of SSAT in proximal tubule epithelial cells, the primary targets of the aforementioned insults, in the mediation of tubular damage and renal dysfunction remains unclear. Our results indicate that the extent of tubular injury is reduced in PT-SSAT-Cko compared to wt mice **(** **Table 1** **;** **Fig. 2b and c** **)**. The potential damaging role of SSAT expression by the parenchymal cells has been demonstrated in transgenic rats, where induction of SSAT in the pancreas leads to the onset of severe pancreatitis [11]; and in hepatocyte specific SSAT-ko mice, where deactivation of SSAT gene in hepatocytes reduces the severity of CCl_4_-induced liver injury [8]. The results presented in this manuscript and studies cited above suggest that the increase in expression of SSAT by parenchymal cells and derangements in polyamine catabolism are important in the mediation of tissue damage and organ dysfunction.

In order to determine the role of SSAT mediated derangements in renal polyamine pools in I/R-mediated kidney injury the polyamine levels in the kidneys of sham-operated and injured wt and PT-SSAT-Cko mice were compared (**Fig. 4**). The only difference in polyamine levels, other than the expected absence of acetylated polyamines in the PT-SSAT-Cko mice, was the significant increase in Put levels in the kidneys of wt mice after I/R injury. The increase in Put levels in the wt but not PT-SSAT-Cko animals is similar to what we reported in our previous studies that examined the role of complete SSAT deficiency on the severity of hepatic and renal I/R injuries [17]. Interestingly, elevated Put levels were shown to reduce the viability of cultured hepatocytes [7]. The stability of renal content of Spd and Spm suggests that polyamine depletion per se may not be the driving force behind the tubular cell injury and renal dysfunction and that enhanced Put levels may play a role in the mediation of tissue injury. It should be noted that polyamine levels were measured in the whole kidney and therefore our results do not reflect the effect of their altered metabolism in a specific group of cells or anatomical segment (e.g. epithelium of the S3 section of the renal proximal tubule that is the primary target of I/R induced injury) of the kidney.

Examination of the mRNA levels of SMO that is up regulated and plays an important role in the mediation of tissue injury [27], [28], in control and injured wt and PT-SSAT-Cko mice revealed that its expression is significantly higher in the kidneys of injured wt compared to PT-SSAT-Cko animals (**Fig. 3b**). The increased expression of SMO in wt but not PT-SSAT-Cko animals as well as the potential cytotoxic effects of the products of polyamine oxidation (e.g. H_2_O_2_ and aminoldahydes) suggest that polyamine oxidation is important in the mediation of I/R induced renal tubular cell injury. The latter was confirmed when it was shown that MDL72527-treated mice had less severe kidney damage after renal I/R injury (**Table 2** **;** **Fig. 5**). These findings show that enhanced polyamine oxidation down stream of increased SSAT expression, most likely via increased expression of toxic metabolites such as H_2_O_2_ and aminoaldehydes, contributes to renal injury. The important role of polyamine oxidation in the mediation of tissue damage is further supported by previous studies which demonstrate that the inhibition of polyamine oxidases or neutralization of the products of their reaction reduces the extent of tissue damage in traumatic, toxic and septic injuries in brain, liver and kidney respectively [6]–[10], [29]–[31].

In order to clarify the mechanism through which enhanced SSAT expression contributes to tissue damage we examined the effect of its induction in cultured cells. We have previously shown that enhanced SSAT expression leads to DNA damage and cell cycle arrest [16]. Our current studies further demonstrate that increased expression of SSAT and up regulation of polyamine degradation lead to the activation of the intrinsic pathway of apoptosis. This data supports previous studies that show increased polyamine catabolism in cultured cells leads to the triggering of apoptosis [32], [33].

The expression of HMGB1, a ligand involved in the activation of innate immune response [22], [34], was also elevated in SSAT-expressing cells (**Fig. 6**). Increased expression of HMGB1 in cells that express high levels of SSAT, as well as reduction in inflammatory cell infiltration in the kidneys of PT-SSAT-Cko mice after I/R injury suggest that down regulation of polyamine catabolism through reduction of cell injury may dampen the activation of innate immune response, there by reducing the severity of organ damage. Therefore, we examined the effect of proximal tubule specific deficiency of SSAT on the activation of innate immune response and onset of apoptosis. The expression of HMGB1, TLR2 and TLR4 were lower and more transient in the injured kidneys of PT-SSAT-Cko mice compared to their wt littermates (**Fig. 7**). Furthermore, leukocyte infiltration, and expression levels of IL-6, MCP-1 and TNF-α, cytokines known to contribute to I/R mediated renal injury [35]–[37], were also reduced in the kidneys of PT-SSAT-Cko- compared to wt-mice (**Fig. 8**). The activation/cleavage of caspase 3 was also lower in the injured kidneys of PT-SSAT-Cko-mice compared to their wt-littermates (**Fig. 9**). These results suggest that the activation of innate immune response and onset of apoptosis after renal I/R injury is less robust in PT-SSAT-Cko- compared to wt-animals. The role of HMGB1, TLR2, TLR4, activation of the innate immune response and onset of apoptosis in the pathogenesis of renal I/R injury are well established [22]–[26]. The expression of HMGB1, TLR2 and TLR4 increases in the kidneys of animals subjected to I/R injury [22]–[26]. In addition, blockade of HMGB1 or deficiency of TLR2 and 4 dampen the inflammatory response (i.e. reduce leukocyte infiltration, lower production of IL-6, MCP-1 and TNF-α, and modulation of apoptotic response) and reduce the severity of renal I/R injury [22]–[26]. Based on our results (**Figs. 6** **–** **8**) we propose that the reduction in the severity of I/R induced kidney injury in PT-SSAT-Cko animals is partially due to the reduction in the initial cell injury which in turn dampens the onset of the innate immune response.

Our previous studies and the results presented here suggest that increased expression of SSAT and enhanced polyamine oxidation in proximal tubule epithelial cells contribute to tubular damage in renal I/R. Furthermore, our cell culture and animal studies suggest that the increase in polyamine catabolism at cellular level and the attendant alterations in cellular polyamine levels play an important role in the initial cell injury. The increased expression of oxidative stress and DNA damage markers, cell cycle arrest, onset of apoptosis and increased expression of HMGB1 in SSAT over-expressing HEK293 but not in un-induced HEK-293 cells supports this hypothesis (**Fig. 6** and reference 16). We also propose that short-circuiting of polyamine catabolism through ablation of SSAT in proximal tubule epithelial cells specifically reduces the severity of initial cellular damage in response to I/R and in turn dampens the activation of the innate immune response further reducing the extent of tubular damage and the severity of renal I/R injury. The latter is supported by our results that show when subjected to renal I/R injury PT-SSAT-Cko animals have reduced neutrophilia, and express reduced levels of HMGB1, TLR2, TLR4 and pro-inflammatory cytokines. The results presented here supports the hypothesis that polyamine back-conversion and oxidation in proximal tubule epithelial cells are important in the pathophysiology of renal I/R injury, and identify novel therapeutic targets and strategies for treatment of I/R induced kidney damage (i.e. SSAT-suppression and/or inhibition of polyamine oxidation).
